# Supplementary material for: High-energy magnetic excitations from heavy quasiparticles in CeCu2Si2
Source: npj Quantum Inf. Author manuscript; Available in PMC 2023 Nov 14. (PMC10644953; doi:10.1038/s41535-021-00358-x)
Supplement: Supp1 [file NIHMS1918313-supplement-Supp1.pdf]

Supplementary Information: High-energy magnetic excitations from heavy quasiparticles in $CeCu_2Si_2$

SUPPLEMENTARY NOTE 1: CALCULATED MAGNETIC EXCITATIONS IN THE (H,K)-PLANE

Since our inelastic neutron scattering measurements are limited to the $[H, H, L]$ scattering plane, we are unable to experimentally probe magnetic excitations along the $(H, -H, 0)$-direction. Nonetheless, our calculations within the random-phase approximation (RPA) suggest that there is indeed modulations along the $(H, -H, 0)$-direction, consistent with the cylindrical (quasi-2D) nature of the well-nested Fermi surface. Supplementary Fig. 1 shows constant-energy slices of the RPA magnetic susceptibility in the $[H, K, 0.5]$-plane, for selected energies from 20 meV to 80 meV. It is clear that in all cases, the magnetic excitations exhibit substantial variations along both $(H, H, 0)$ and $(H, -H, 0)$. On the other hand, little or no L-modulation is present in our calculations. The magnetic susceptibility of $CeCu_2Si_2$ from our RPA calculations therefore evidence quasi-2D magnetic excitations, consistent with our experimental findings.

SUPPLEMENTARY NOTE 2: SHIFT OF SPECTRAL WEIGHT IN CONSTANT-ENERGY SCANS

The subtle shift of spectral weight in momentum between the normal and superconducting states shown in Fig. 4 of the main text results from gapping of electronic states near the Fermi level. As schematically shown in Supplemental Fig. 3(a), for the normal state band with dispersion $E_{\mathrm{N}}(k)$, a particle-hole excitation connecting k_1 and k_2 has an energy $E_{\mathrm{N}}(k_2) - E_{\mathrm{N}}(k_1)$. When a gap 2Δ opens at low temperatures, the electronic states at k_1 and k_2 are pushed away from the Fermi level so that $E_{\mathrm{S}}(k_1) < E_{\mathrm{N}}(k_1)$ and $E_{\mathrm{S}}(k_2) > E_{\mathrm{N}}(k_1)$. The particle-hole excitations connecting k_1 and k_2 now has an energy $E_{\mathrm{S}}(k_2) - E_{\mathrm{S}}(k_1) > E_{\mathrm{N}}(k_2) - E_{\mathrm{N}}(k_1)$. Since this is generally true for arbitrary choices of k_1 and k_2, the magnetic excitations that arise from these particle-hole excitations effectively move up in energy once a gap is introduced. Such an effect is most prominent for magnetic excitations $\lesssim 2\Delta$, and becomes less important with increasing energy. This shift of magnetic spectral weight to higher energies combined with the asymmetric dispersion of magnetic excitations results in a shift of spectral weight towards smaller momentum in constant-energy measurements, as schematically depicted in Supplemental Fig. 3(b). The observation of such a subtle shift in turn also supports the description of magnetic excitations in $CeCu_2Si_2$ as particle-hole excitations.

Disclaimer: The identification of any commercial product or trade name does not imply endorsement or recommendation by the National Institute of Standards and Technology.

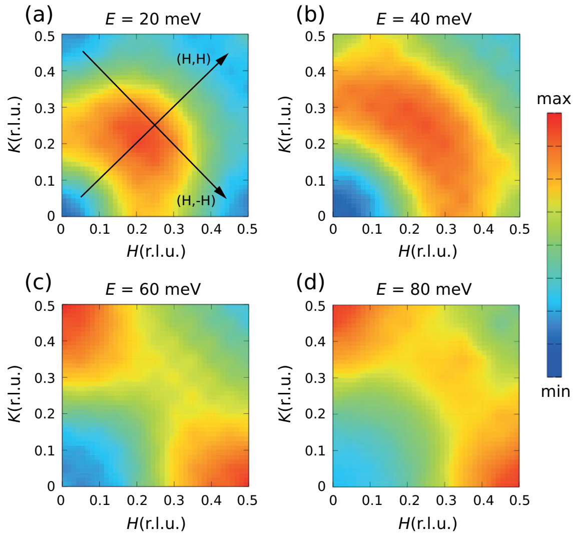

Supplementary Figure 1: Constant-energy slices of the RPA magnetic susceptibility in the $(H, K, 0.5)$-plane for (a) $E = 20$ meV, (b) $E = 40$ meV, (c) $E = 60$ meV and (d) $E = 80$ meV. As our inelastic neutron scattering measurements are restricted to the $[H, H, L]$-plane, our experiments probe magnetic excitations along the (H, H)-direction, but not the $(H, -H)$-direction.

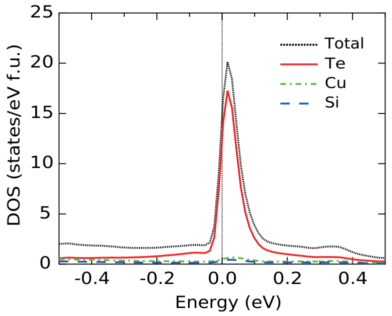

Supplementary Figure 2: The partial density of states of $CeCu_2Si_2$ from LDA+U calculations.

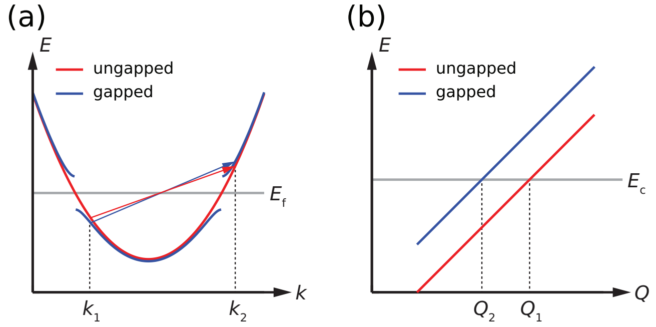

Supplementary Figure 3: (a) Schematic demonstration of the opening of a gap at the Fermi level leads to particle-hole excitations connecting k_1 and k_2 having a larger energy. (b) Schematic demonstrating that the opening of a gap at the Fermi level leads to a shift of magnetic excitations to a smaller momentum in constant-energy scans at $E = E_c$. Magnetic excitations in the gapped state intersects E_c at a smaller momentum (Q_2) compared to magnetic excitations in the ungapped state (Q_1).

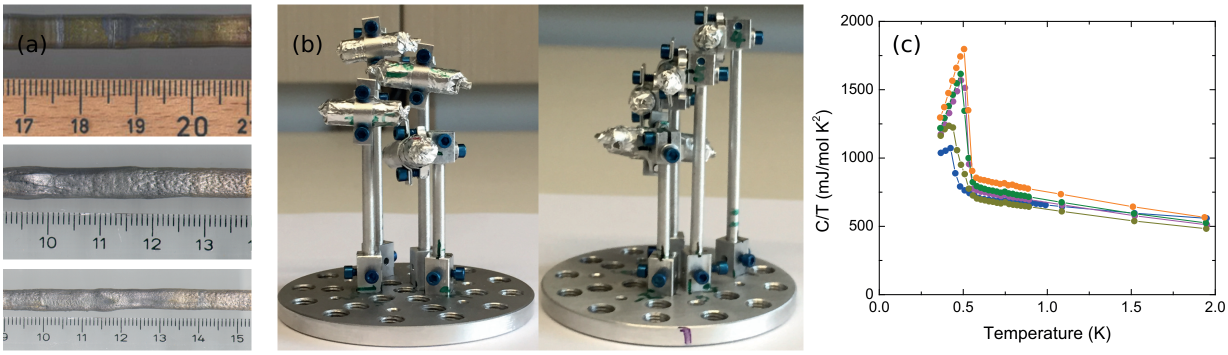

Supplementary Figure 4: (a) Rod-shaped $CeCu_2Si_2$ single crystal samples with lengths up to several centimeters and radii around 5 mm. (b) Co-aligned $CeCu_2Si_2$ samples. (c) Specific heat $C(T)/T$ measured for several pieces of our $CeCu_2Si_2$ samples.

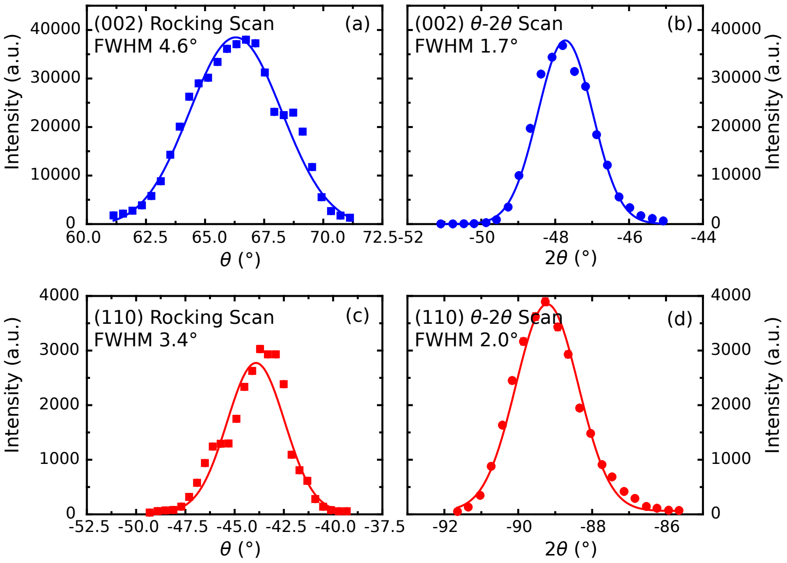

Supplementary Figure 5: (a) (002) rocking scan. (b) (002) $\theta - 2\theta$ scan. (c) (110) rocking scan. (d) (110) $\theta - 2\theta$ scan.

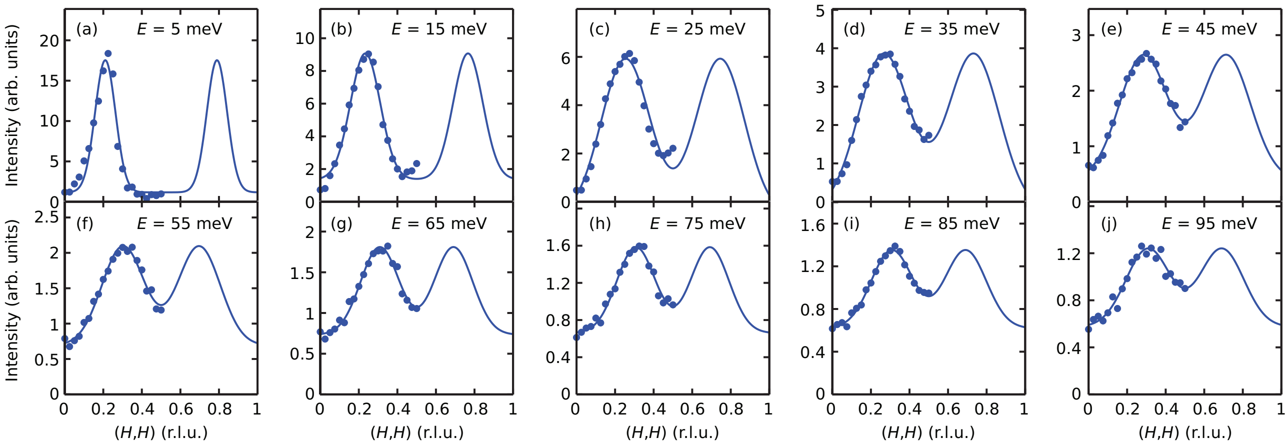

Supplementary Figure 6: Calculated magnetic susceptibilities along (H, H) for different energies. The solid lines are fits to two Gaussian peaks.

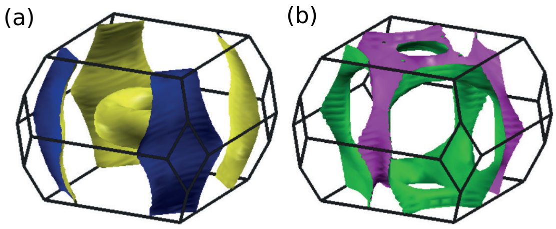

Supplementary Figure 7: The Fermi surfaces in our LDA+U calculations. (a) The heavy electron Fermi surface sheet and (b) the complex hole Fermi surface sheet.

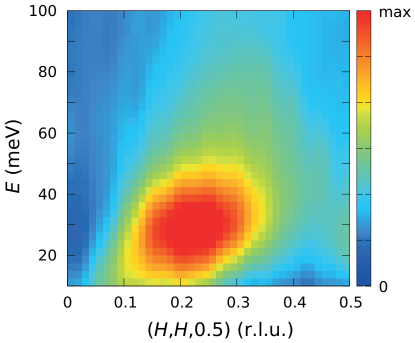

Supplementary Figure 8: The bare magnetic susceptibility from our LDA+U band structure.
